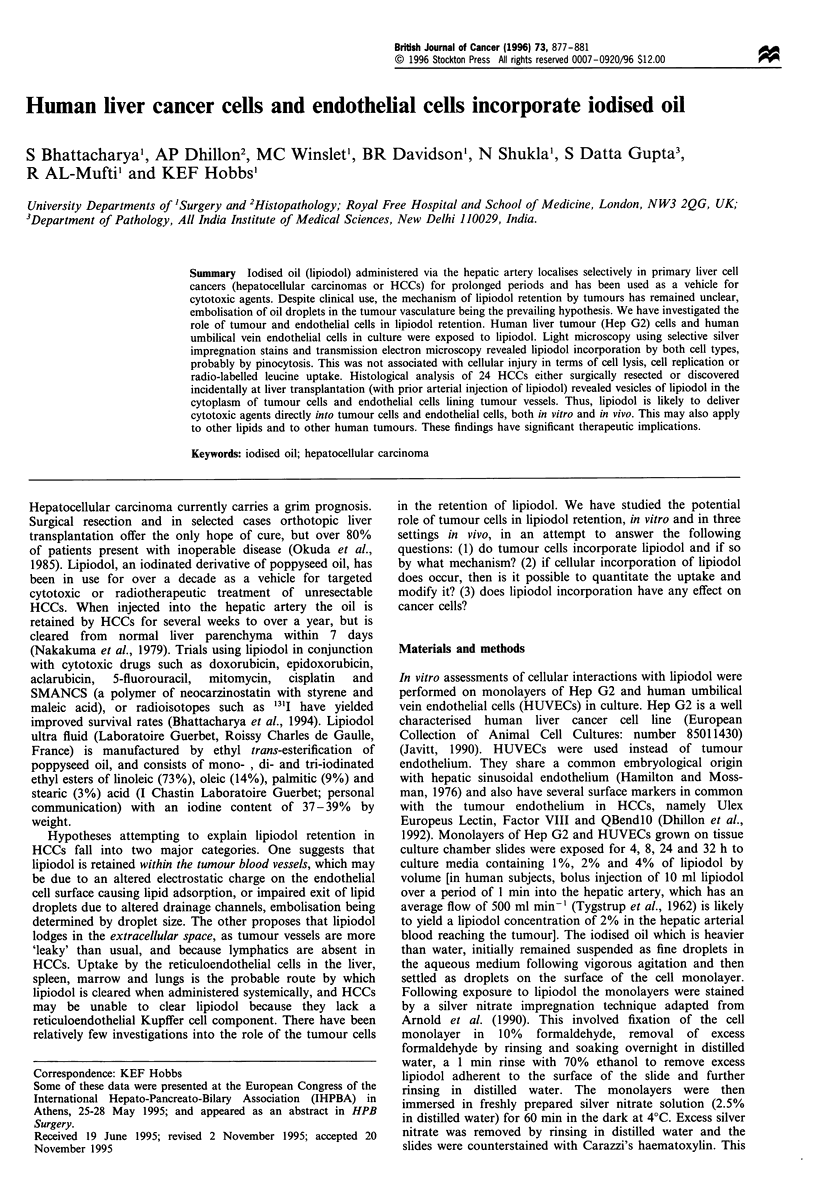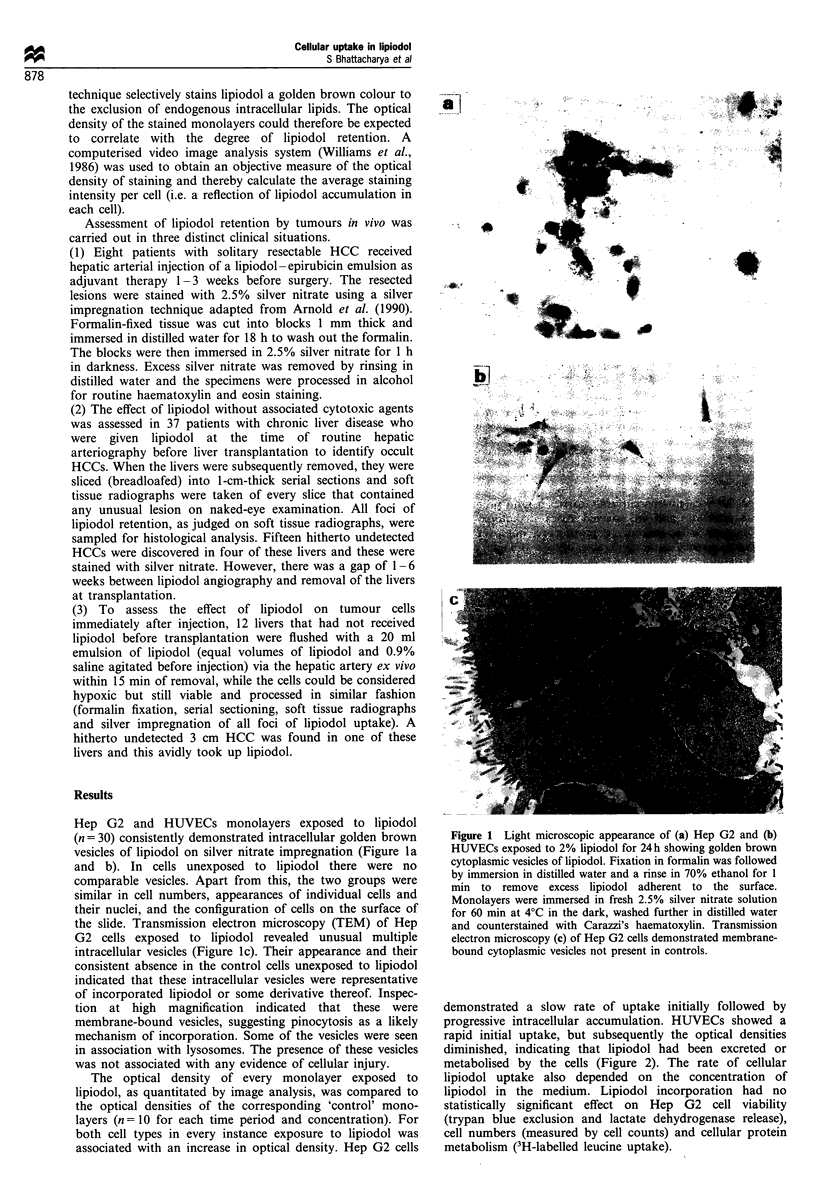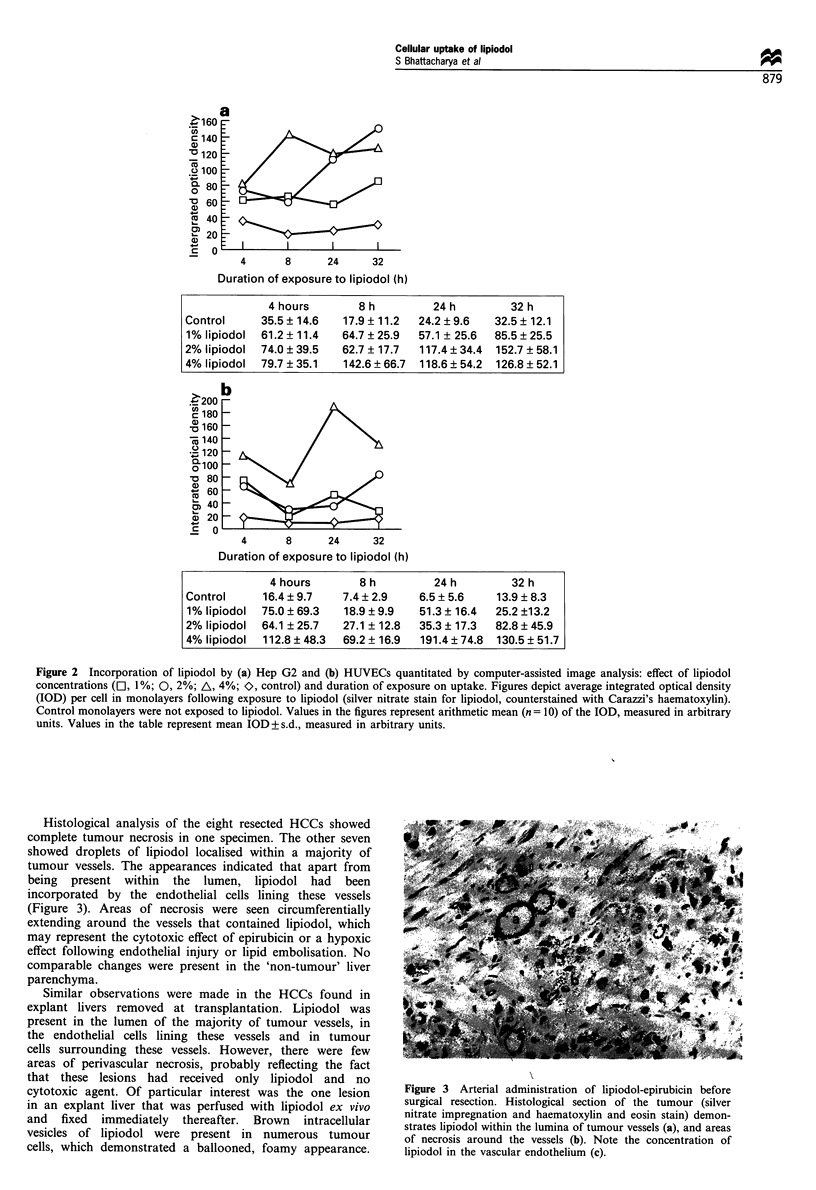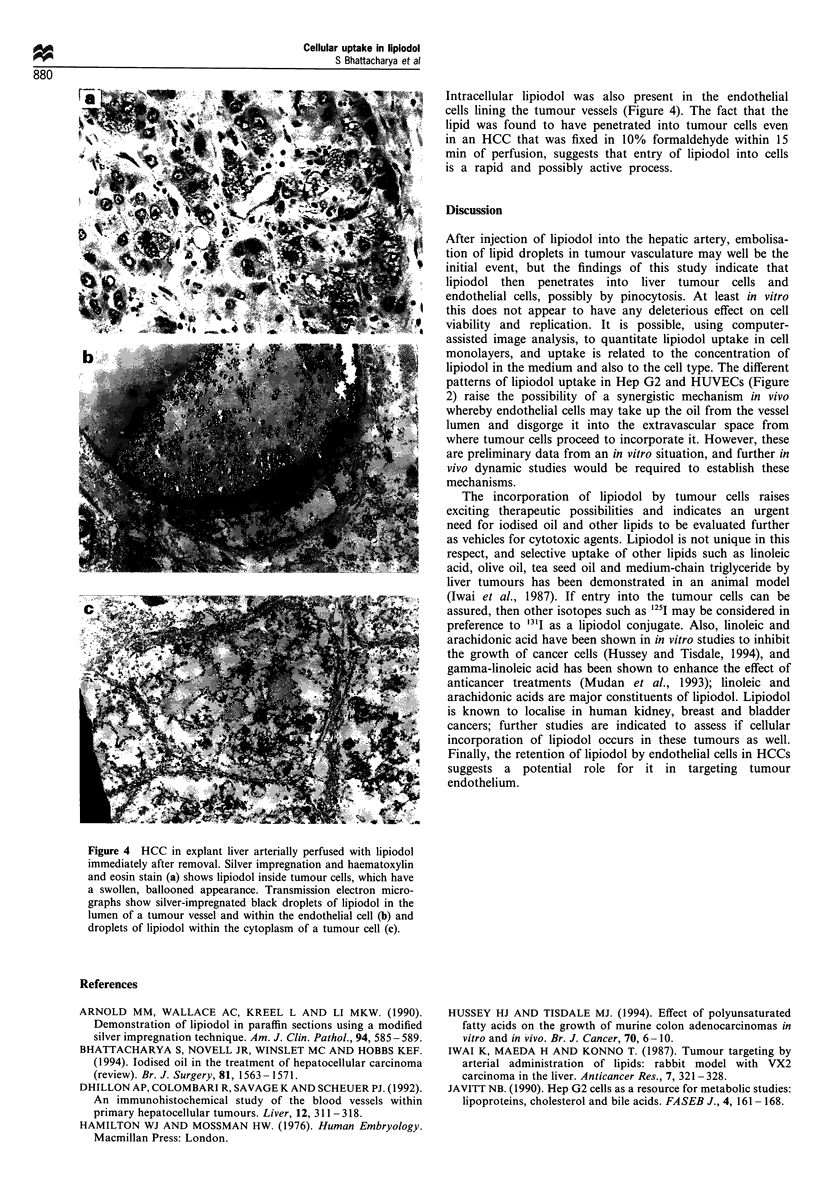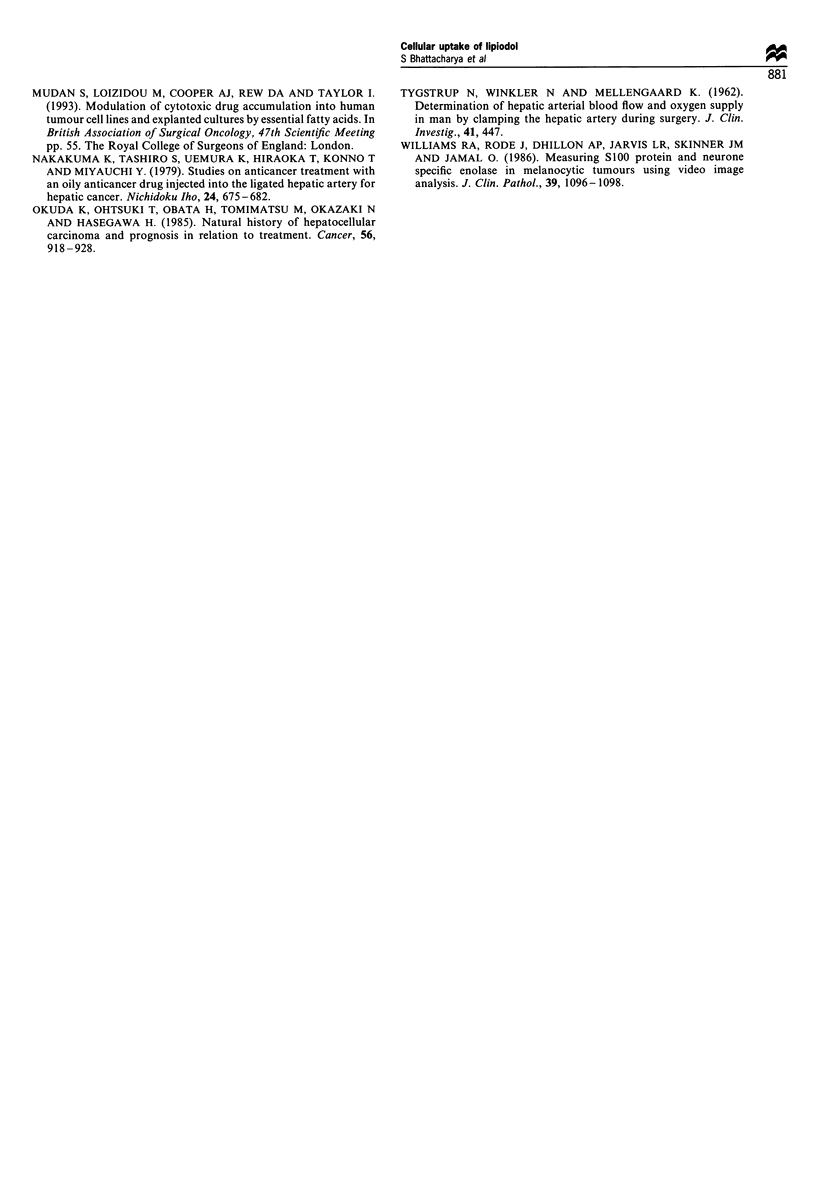# Human liver cancer cells and endothelial cells incorporate iodised oil.

**DOI:** 10.1038/bjc.1996.156

**Published:** 1996-04

**Authors:** S. Bhattacharya, A. P. Dhillon, M. C. Winslet, B. R. Davidson, N. Shukla, S. D. Gupta, R. Al-Mufti, K. E. Hobbs

**Affiliations:** University Department of Surgery, Royal Free Hospital and School of Medicine, London, UK.

## Abstract

**Images:**


British Journal of Cancer (1996) 73, 877-881
© 1996 Stockton Press All rights reserved 0007-0920/96 $12.00

### Human liver cancer cells and endothelial cells incorporate iodised oil

S Bhattacharya[1], AP Dhillon[2], MC Winslet[1], BR Davidson[1], N Shukla[1], S Datta Gupta[3], R AL-Mufti[1] and KEF Hobbs[1]

*University Departments of [1]Surgery and [2]Histopathology; Royal Free Hospital and School of Medicine, London, NW3 2QG, UK; [3]Department of Pathology, All India Institute of Medical Sciences, New Delhi 110029, India.*

**Summary** Iodised oil (lipiodol) administered via the hepatic artery localises selectively in primary liver cell cancers (hepatocellular carcinomas or HCCs) for prolonged periods and has been used as a vehicle for cytotoxic agents. Despite clinical use, the mechanism of lipiodol retention by tumours has remained unclear, embolisation of oil droplets in the tumour vasculature being the prevailing hypothesis. We have investigated the role of tumour and endothelial cells in lipiodol retention. Human liver tumour (Hep G2) cells and human umbilical vein endothelial cells in culture were exposed to lipiodol. Light microscopy using selective silver impregnation stains and transmission electron microscopy revealed lipiodol incorporation by both cell types, probably by pinocytosis. This was not associated with cellular injury in terms of cell lysis, cell replication or radio-labelled leucine uptake. Histological analysis of 24 HCCs either surgically resected or discovered incidentally at liver transplantation (with prior arterial injection of lipiodol) revealed vesicles of lipiodol in the cytoplasm of tumour cells and endothelial cells lining tumour vessels. Thus, lipiodol is likely to deliver cytotoxic agents directly *into* tumour cells and endothelial cells, both *in vitro* and *in vivo*. This may also apply to other lipids and to other human tumours. These findings have significant therapeutic implications.

**Keywords:** iodised oil; hepatocellular carcinoma

Hepatocellular carcinoma currently carries a grim prognosis. Surgical resection and in selected cases orthotopic liver transplantation offer the only hope of cure, but over 80% of patients present with inoperable disease (Okuda *et al.*, 1985). Lipiodol, an iodinated derivative of poppyseed oil, has been in use for over a decade as a vehicle for targeted cytotoxic or radiotherapeutic treatment of unresectable HCCs. When injected into the hepatic artery the oil is retained by HCCs for several weeks to over a year, but is cleared from normal liver parenchyma within 7 days (Nakakuma *et al.*, 1979). Trials using lipiodol in conjunction with cytotoxic drugs such as doxorubicin, epidoxorubicin, aclarubicin, 5-fluorouracil, mitomycin, cisplatin and SMANCS (a polymer of neocarzinostatin with styrene and maleic acid), or radioisotopes such as $^{131}I$ have yielded improved survival rates (Bhattacharya *et al.*, 1994). Lipiodol ultra fluid (Laboratoire Guerbet, Roissy Charles de Gaulle, France) is manufactured by ethyl *trans*-esterification of poppyseed oil, and consists of mono- , di- and tri-iodinated ethyl esters of linoleic (73%), oleic (14%), palmitic (9%) and stearic (3%) acid (I Chastin Laboratoire Guerbet; personal communication) with an iodine content of 37–39% by weight.

Hypotheses attempting to explain lipiodol retention in HCCs fall into two major categories. One suggests that lipiodol is retained *within the tumour blood vessels*, which may be due to an altered electrostatic charge on the endothelial cell surface causing lipid adsorption, or impaired exit of lipid droplets due to altered drainage channels, embolisation being determined by droplet size. The other proposes that lipiodol lodges in the *extracellular space*, as tumour vessels are more 'leaky' than usual, and because lymphatics are absent in HCCs. Uptake by the reticuloendothelial cells in the liver, spleen, marrow and lungs is the probable route by which lipiodol is cleared when administered systemically, and HCCs may be unable to clear lipiodol because they lack a reticuloendothelial Kupffer cell component. There have been relatively few investigations into the role of the tumour cells in the retention of lipiodol. We have studied the potential role of tumour cells in lipiodol retention, *in vitro* and in three settings *in vivo*, in an attempt to answer the following questions: (1) do tumour cells incorporate lipiodol and if so by what mechanism? (2) if cellular incorporation of lipiodol does occur, then is it possible to quantitate the uptake and modify it? (3) does lipiodol incorporation have any effect on cancer cells?

### Materials and methods

*In vitro* assessments of cellular interactions with lipiodol were performed on monolayers of Hep G2 and human umbilical vein endothelial cells (HUVECs) in culture. Hep G2 is a well characterised human liver cancer cell line (European Collection of Animal Cell Cultures: number 85011430) (Javitt, 1990). HUVECs were used instead of tumour endothelium. They share a common embryological origin with hepatic sinusoidal endothelium (Hamilton and Mossman, 1976) and also have several surface markers in common with the tumour endothelium in HCCs, namely Ulex Europeus Lectin, Factor VIII and QBend10 (Dhillon *et al.*, 1992). Monolayers of Hep G2 and HUVECs grown on tissue culture chamber slides were exposed for 4, 8, 24 and 32 h to culture media containing 1%, 2% and 4% of lipiodol by volume [in human subjects, bolus injection of 10 ml lipiodol over a period of 1 min into the hepatic artery, which has an average flow of 500 ml $min^{-1}$ (Tygstrup *et al.*, 1962) is likely to yield a lipiodol concentration of 2% in the hepatic arterial blood reaching the tumour]. The iodised oil which is heavier than water, initially remained suspended as fine droplets in the aqueous medium following vigorous agitation and then settled as droplets on the surface of the cell monolayer. Following exposure to lipiodol the monolayers were stained by a silver nitrate impregnation technique adapted from Arnold *et al.* (1990). This involved fixation of the cell monolayer in 10% formaldehyde, removal of excess formaldehyde by rinsing and soaking overnight in distilled water, a 1 min rinse with 70% ethanol to remove excess lipiodol adherent to the surface of the slide and further rinsing in distilled water. The monolayers were then immersed in freshly prepared silver nitrate solution (2.5% in distilled water) for 60 min in the dark at 4°C. Excess silver nitrate was removed by rinsing in distilled water and the slides were counterstained with Carazzi's haematoxylin. This

Correspondence: KEF Hobbs
Some of these data were presented at the European Congress of the International Hepato-Pancreato-Bilary Association (IHPBA) in Athens, 25-28 May 1995; and appeared as an abstract in *HPB Surgery*.
Received 19 June 1995; revised 2 November 1995; accepted 20 November 1995

technique selectively stains lipiodol a golden brown colour to the exclusion of endogenous intracellular lipids. The optical density of the stained monolayers could therefore be expected to correlate with the degree of lipiodol retention. A computerised video image analysis system (Williams et al., 1986) was used to obtain an objective measure of the optical density of staining and thereby calculate the average staining intensity per cell (i.e. a reflection of lipiodol accumulation in each cell).

Assessment of lipiodol retention by tumours *in vivo* was carried out in three distinct clinical situations.

(1) Eight patients with solitary resectable HCC received hepatic arterial injection of a lipiodol–epirubicin emulsion as adjuvant therapy 1–3 weeks before surgery. The resected lesions were stained with 2.5% silver nitrate using a silver impregnation technique adapted from Arnold *et al.* (1990). Formalin-fixed tissue was cut into blocks 1 mm thick and immersed in distilled water for 18 h to wash out the formalin. The blocks were then immersed in 2.5% silver nitrate for 1 h in darkness. Excess silver nitrate was removed by rinsing in distilled water and the specimens were processed in alcohol for routine haematoxylin and eosin staining.

(2) The effect of lipiodol without associated cytotoxic agents was assessed in 37 patients with chronic liver disease who were given lipiodol at the time of routine hepatic arteriography before liver transplantation to identify occult HCCs. When the livers were subsequently removed, they were sliced (breadloafed) into 1-cm-thick serial sections and soft tissue radiographs were taken of every slice that contained any unusual lesion on naked-eye examination. All foci of lipiodol retention, as judged on soft tissue radiographs, were sampled for histological analysis. Fifteen hitherto undetected HCCs were discovered in four of these livers and these were stained with silver nitrate. However, there was a gap of 1–6 weeks between lipiodol angiography and removal of the livers at transplantation.

(3) To assess the effect of lipiodol on tumour cells immediately after injection, 12 livers that had not received lipiodol before transplantation were flushed with a 20 ml emulsion of lipiodol (equal volumes of lipiodol and 0.9% saline agitated before injection) via the hepatic artery *ex vivo* within 15 min of removal, while the cells could be considered hypoxic but still viable and processed in similar fashion (formalin fixation, serial sectioning, soft tissue radiographs and silver impregnation of all foci of lipiodol uptake). A hitherto undetected 3 cm HCC was found in one of these livers and this avidly took up lipiodol.

### Results

Hep G2 and HUVECs monolayers exposed to lipiodol ($n = 30$) consistently demonstrated intracellular golden brown vesicles of lipiodol on silver nitrate impregnation (Figure 1a and b). In cells unexposed to lipiodol there were no comparable vesicles. Apart from this, the two groups were similar in cell numbers, appearances of individual cells and their nuclei, and the configuration of cells on the surface of the slide. Transmission electron microscopy (TEM) of Hep G2 cells exposed to lipiodol revealed unusual multiple intracellular vesicles (Figure 1c). Their appearance and their consistent absence in the control cells unexposed to lipiodol indicated that these intracellular vesicles were representative of incorporated lipiodol or some derivative thereof. Inspection at high magnification indicated that these were membrane-bound vesicles, suggesting pinocytosis as a likely mechanism of incorporation. Some of the vesicles were seen in association with lysosomes. The presence of these vesicles was not associated with any evidence of cellular injury.

The optical density of every monolayer exposed to lipiodol, as quantitated by image analysis, was compared to the optical densities of the corresponding 'control' monolayers ($n = 10$ for each time period and concentration). For both cell types in every instance exposure to lipiodol was associated with an increase in optical density. Hep G2 cells

**Figure 1** Light microscopic appearance of (**a**) Hep G2 and (**b**) HUVECs exposed to 2% lipiodol for 24 h showing golden brown cytoplasmic vesicles of lipiodol. Fixation in formalin was followed by immersion in distilled water and a rinse in 70% ethanol for 1 min to remove excess lipiodol adherent to the surface. Monolayers were immersed in fresh 2.5% silver nitrate solution for 60 min at 4°C in the dark, washed further in distilled water and counterstained with Carazzi's haematoxylin. Transmission electron microscopy (**c**) of Hep G2 cells demonstrated membrane-bound cytoplasmic vesicles not present in controls.

demonstrated a slow rate of uptake initially followed by progressive intracellular accumulation. HUVECs showed a rapid initial uptake, but subsequently the optical densities diminished, indicating that lipiodol had been excreted or metabolised by the cells (Figure 2). The rate of cellular lipiodol uptake also depended on the concentration of lipiodol in the medium. Lipiodol incorporation had no statistically significant effect on Hep G2 cell viability (trypan blue exclusion and lactate dehydrogenase release), cell numbers (measured by cell counts) and cellular protein metabolism ($^3$H-labelled leucine uptake).

| | 4 hours | 8 h | 24 h | 32 h |
|---|---|---|---|---|
| Control | 35.5 ± 14.6 | 17.9 ± 11.2 | 24.2 ± 9.6 | 32.5 ± 12.1 |
| 1% lipiodol | 61.2 ± 11.4 | 64.7 ± 25.9 | 57.1 ± 25.6 | 85.5 ± 25.5 |
| 2% lipiodol | 74.0 ± 39.5 | 62.7 ± 17.7 | 117.4 ± 34.4 | 152.7 ± 58.1 |
| 4% lipiodol | 79.7 ± 35.1 | 142.6 ± 66.7 | 118.6 ± 54.2 | 126.8 ± 52.1 |

| | 4 hours | 8 h | 24 h | 32 h |
|---|---|---|---|---|
| Control | 16.4 ± 9.7 | 7.4 ± 2.9 | 6.5 ± 5.6 | 13.9 ± 8.3 |
| 1% lipiodol | 75.0 ± 69.3 | 18.9 ± 9.9 | 51.3 ± 16.4 | 25.2 ±13.2 |
| 2% lipiodol | 64.1 ± 25.7 | 27.1 ± 12.8 | 35.3 ± 17.3 | 82.8 ± 45.9 |
| 4% lipiodol | 112.8 ± 48.3 | 69.2 ± 16.9 | 191.4 ± 74.8 | 130.5 ± 51.7 |

**Figure 2** Incorporation of lipiodol by (**a**) Hep G2 and (**b**) HUVECs quantitated by computer-assisted image analysis: effect of lipiodol concentrations (□, 1%; ○, 2%; △, 4%; ◇, control) and duration of exposure on uptake. Figures depict average integrated optical density (IOD) per cell in monolayers following exposure to lipiodol (silver nitrate stain for lipiodol, counterstained with Carazzi's haematoxylin). Control monolayers were not exposed to lipiodol. Values in the figures represent arithmetic mean ($n = 10$) of the IOD, measured in arbitrary units. Values in the table represent mean IOD ± s.d., measured in arbitrary units.

Histological analysis of the eight resected HCCs showed complete tumour necrosis in one specimen. The other seven showed droplets of lipiodol localised within a majority of tumour vessels. The appearances indicated that apart from being present within the lumen, lipiodol had been incorporated by the endothelial cells lining these vessels (Figure 3). Areas of necrosis were seen circumferentially extending around the vessels that contained lipiodol, which may represent the cytotoxic effect of epirubicin or a hypoxic effect following endothelial injury or lipid embolisation. No comparable changes were present in the 'non-tumour' liver parenchyma.

Similar observations were made in the HCCs found in explant livers removed at transplantation. Lipiodol was present in the lumen of the majority of tumour vessels, in the endothelial cells lining these vessels and in tumour cells surrounding these vessels. However, there were few areas of perivascular necrosis, probably reflecting the fact that these lesions had received only lipiodol and no cytotoxic agent. Of particular interest was the one lesion in an explant liver that was perfused with lipiodol *ex vivo* and fixed immediately thereafter. Brown intracellular vesicles of lipiodol were present in numerous tumour cells, which demonstrated a ballooned, foamy appearance.

**Figure 3** Arterial administration of lipiodol-epirubicin before surgical resection. Histological section of the tumour (silver nitrate impregnation and haematoxylin and eosin stain) demonstrates lipiodol within the lumina of tumour vessels (**a**), and areas of necrosis around the vessels (**b**). Note the concentration of lipiodol in the vascular endothelium (**c**).

**Figure 4** HCC in explant liver arterially perfused with lipiodol immediately after removal. Silver impregnation and haematoxylin and eosin stain (**a**) shows lipiodol inside tumour cells, which have a swollen, ballooned appearance. Transmission electron micrographs show silver-impregnated black droplets of lipiodol in the lumen of a tumour vessel and within the endothelial cell (**b**) and droplets of lipiodol within the cytoplasm of a tumour cell (**c**).

Intracellular lipiodol was also present in the endothelial cells lining the tumour vessels (Figure 4). The fact that the lipid was found to have penetrated into tumour cells even in an HCC that was fixed in 10% formaldehyde within 15 min of perfusion, suggests that entry of lipiodol into cells is a rapid and possibly active process.

### Discussion

After injection of lipiodol into the hepatic artery, embolisation of lipid droplets in tumour vasculature may well be the initial event, but the findings of this study indicate that lipiodol then penetrates into liver tumour cells and endothelial cells, possibly by pinocytosis. At least *in vitro* this does not appear to have any deleterious effect on cell viability and replication. It is possible, using computer-assisted image analysis, to quantitate lipiodol uptake in cell monolayers, and uptake is related to the concentration of lipiodol in the medium and also to the cell type. The different patterns of lipiodol uptake in Hep G2 and HUVECs (Figure 2) raise the possibility of a synergistic mechanism *in vivo* whereby endothelial cells may take up the oil from the vessel lumen and disgorge it into the extravascular space from where tumour cells proceed to incorporate it. However, these are preliminary data from an *in vitro* situation, and further *in vivo* dynamic studies would be required to establish these mechanisms.

The incorporation of lipiodol by tumour cells raises exciting therapeutic possibilities and indicates an urgent need for iodised oil and other lipids to be evaluated further as vehicles for cytotoxic agents. Lipiodol is not unique in this respect, and selective uptake of other lipids such as linoleic acid, olive oil, tea seed oil and medium-chain triglyceride by liver tumours has been demonstrated in an animal model (Iwai *et al.*, 1987). If entry into the tumour cells can be assured, then other isotopes such as $^{125}I$ may be considered in preference to $^{131}I$ as a lipiodol conjugate. Also, linoleic and arachidonic acid have been shown in *in vitro* studies to inhibit the growth of cancer cells (Hussey and Tisdale, 1994), and gamma-linoleic acid has been shown to enhance the effect of anticancer treatments (Mudan *et al.*, 1993); linoleic and arachidonic acids are major constituents of lipiodol. Lipiodol is known to localise in human kidney, breast and bladder cancers; further studies are indicated to assess if cellular incorporation of lipiodol occurs in these tumours as well. Finally, the retention of lipiodol by endothelial cells in HCCs suggests a potential role for it in targeting tumour endothelium.

### References

ARNOLD MM, WALLACE AC, KREEL L AND LI MKW. (1990). Demonstration of lipiodol in paraffin sections using a modified silver impregnation technique. *Am. J. Clin. Pathol.*, **94**, 585–589.

BHATTACHARYA S, NOVELL JR, WINSLET MC AND HOBBS KEF. (1994). Iodised oil in the treatment of hepatocellular carcinoma (review). *Br. J. Surgery*, **81**, 1563–1571.

DHILLON AP, COLOMBARI R, SAVAGE K AND SCHEUER PJ. (1992). An immunohistochemical study of the blood vessels within primary hepatocellular tumours. *Liver*, **12**, 311–318.

HAMILTON WJ AND MOSSMAN HW. (1976). *Human Embryology*. Macmillan Press: London.

HUSSEY HJ AND TISDALE MJ. (1994). Effect of polyunsaturated fatty acids on the growth of murine colon adenocarcinomas *in vitro* and *in vivo*. *Br. J. Cancer*, **70**, 6–10.

IWAI K, MAEDA H AND KONNO T. (1987). Tumour targeting by arterial administration of lipids: rabbit model with VX2 carcinoma in the liver. *Anticancer Res.*, **7**, 321–328.

JAVITT NB. (1990). Hep G2 cells as a resource for metabolic studies: lipoproteins, cholesterol and bile acids. *FASEB J.*, **4**, 161–168.

MUDAN S, LOIZIDOU M, COOPER AJ, REW DA AND TAYLOR I. (1993). Modulation of cytotoxic drug accumulation into human tumour cell lines and explanted cultures by essential fatty acids. In *British Association of Surgical Oncology, 47th Scientific Meeting* pp. 55. The Royal College of Surgeons of England: London.

NAKAKUMA K, TASHIRO S, UEMURA K, HIRAOKA T, KONNO T AND MIYAUCHI Y. (1979). Studies on anticancer treatment with an oily anticancer drug injected into the ligated hepatic artery for hepatic cancer. *Nichidoku Iho*, **24**, 675–682.

OKUDA K, OHTSUKI T, OBATA H, TOMIMATSU M, OKAZAKI N AND HASEGAWA H. (1985). Natural history of hepatocellular carcinoma and prognosis in relation to treatment. *Cancer*, **56**, 918–928.

TYGSTRUP N, WINKLER N AND MELLENGAARD K. (1962). Determination of hepatic arterial blood flow and oxygen supply in man by clamping the hepatic artery during surgery. *J. Clin. Investig.*, **41**, 447.

WILLIAMS RA, RODE J, DHILLON AP, JARVIS LR, SKINNER JM AND JAMAL O. (1986). Measuring S100 protein and neurone specific enolase in melanocytic tumours using video image analysis. *J. Clin. Pathol.*, **39**, 1096–1098.